# Association between genetic polymorphisms in the autophagy-related 5 gene promoter and the risk of sepsis

**DOI:** 10.1038/s41598-017-09978-5

**Published:** 2017-08-24

**Authors:** Yiming Shao, Feng Chen, Yuhua Chen, Wenying Zhang, Yao Lin, Yujie Cai, Zihan Yin, Shoubao Tao, Qinghui Liao, Jianghao Zhao, Hui Mai, Yanfang He, Junbing He, Lili Cui

**Affiliations:** 1 The Intensive Care Unit, Guangdong Key Laboratory of Age-Related Cardiac and Cerebral Diseases, Affiliated Hospital of Guangdong Medical University, Zhanjiang, Guangdong P.R. China; 2 Institute of Neurology, Guangdong Key Laboratory of Age-Related Cardiac and Cerebral Diseases, Affiliated Hospital of Guangdong Medical University, Zhanjiang, Guangdong P.R. China; 3 The Department of Endocrinology and Metabolism, Longgang District People’s Hospital of Shenzhen, Shenzhen, Guangdong P.R. China; 4 The Department of Stomatology, Guangdong Key Laboratory of Age-Related Cardiac and Cerebral Diseases, Affiliated Hospital of Guangdong Medical University, Zhanjiang, Guangdong P.R. China

## Abstract

Previous studies demonstrated significant roles of autophagy in the pathogenesis of sepsis, but few studies focused on the effect of autophagy-related SNPs on sepsis susceptibility. In this present study, five polymorphisms of ATG5/ATG16L1 were investigated for the possible risk on sepsis in a Chinese Han population. Our results showed that ATG5 expression levels decreased with the severity of sepsis, and rs506027 T > C and rs510432 G > A were associated with sepsis progression and mortality. Moreover, the rs506027 TT and rs510432 GG carriers also exhibited increased expression levels of ATG5. Functional assays showed that ATG5 knockdown elevated the secretion of pro-inflammatory cytokines in THP-1 cells, and the extracted mononuclear cell of the risk C-A carriers exhibited decreased ATG5 expression levels, leading to enhanced releases of TNF-α and IL-1β under LPS stimulation *in vitro*. Furthermore, ATG5 T-G haplotype mutation showed higher promoter activities compared to C-A haplotype mutation, suggesting the effect of these SNPs on ATG5 gene transcription. Taken together, these results above indicated that these two ATG5 promoter polymorphisms may be functional and clinically significant for sepsis progression, underscoring its potentially therapeutic implications for sepsis and other inflammatory diseases.

## Introduction

Sepsis is a complex clinical syndrome resulting from a detrimental systemic inflammatory response to the microbe infection^1, 2^, it may result in multiple organ failure and progression to septic shock, which is correlated with poor disease outcomes^3^. Increasing evidence has implicated several genetic polymorphisms of genes associated with inflammatory and immune responses in the pathogenesis of sepsis^4–6^. Genetic sequence identification and association between genotypes and different immunological profiles or disease outcomes may contribute to the genetic diagnosis and interventional treatment of sepsis, which may largely improve the prognosis of septic patients^7, 8^.

Autophagy is a cytoprotective process that regulates the degradation of long-lived proteins and organelles, such as mitochondria, and the removal of intracellular microorganisms^9, 10^. Autophagy also plays significant roles in innate and adaptive immune responses of inflammation-related diseases, such as sepsis^11–14^. Among the proteins that are implicated in autophagy initiation and regulation, autophagy-related 5 (ATG5) is part of a large protein complex together with autophagy-related 16-like 1 (ATG16L1) and ATG12, and ATG5 plays vital roles in the maturation of the autophagosome by promoting autophagosome expansion^15^. Autophagy is involved in the evolving battle between the host and infecting microbes that influences pathogenesis and infection outcome^16, 17^. Several studies have indicated that ATG5 is vital for resistance to *Mycobacterium tuberculosis* by preventing the immunopathology mediated by polymorphonuclear neutrophils^18, 19^. The ATG16L1 hypomorphic mouse, with decreased autophagy activity, was clearly associated with predisposition to lethality in pneumonia and sepsis models of *Staphylococcus aureus* infection^20^. It have also been demonstrated that macrophages deficient in the autophagy proteins ATG5 and ATG16L1 showed markedly higher production of pro-inflammatory cytokines after lipopolysaccharide stimulation^21, 22^, and defects in these autophagy-related proteins was correlated with an increased septic response and lethality in murine modes of sepsis induced by cecal ligation puncture^23–25^. These lines of evidence demonstrate that ATG5 and ATG16L1, which have been implicated in autophagy initiation and regulation, play a significant role in the pathogenic mechanism underlying the development of sepsis. The human ATG5 gene is located on chromosome 6q21 and encodes a protein of 276 amino acids^26^. A growing number of studies indicated that several ATG5 promoter polymorphisms, including rs510432 and rs506027, were involved in various inflammatory diseases such as asthma, Behcet’s disease and systemic lupus erythematosus^27–29^. With regards to ATG16L1, which was verified as a disease-susceptibility gene for Crohn’s disease, rs10210302 and rs2241880 SNPs have also been reported to be associated with *Candida* infections and colorectal cancer^30–32^, supporting that these autophagy-related SNPs may have potential function to confer the susceptibility of inflammation-related disease.

Given the evidence suggesting that ATG5 and ATG16L1 significantly affected the pathogenic mechanism and development of sepsis and several related SNPs contributed to the risk of inflammatory diseases, we carried out this hospital-based case-control study to ascertain whether the genetic polymorphisms of ATG5 (rs510432, rs506027 and rs548234) and ATG16L1 (rs10210302 and rs2241880) were associated with sepsis in a Chinese Han population. Moreover, functional assays of these genetic polymorphisms were also explored *in vitro* to evaluate the possible associations between these polymorphisms and sepsis.

## Results

### Clinical data

The demographic characteristics for the 803 studied subjects (403 sepsis patients and 400 healthy volunteers) were shown in Table 1. No significant differences were found between the sepsis cases and controls in regard to age (P = 0.110) or gender (P = 0.323). Then the 403 sepsis cases were separated into three subgroups by sepsis severity on the basis of the International Sepsis Definitions Conference^33, 34^, as follows: mild sepsis (74), severe sepsis (191) and septic shock (138). The major sources of the infection were lung (63.0%), abdomen (23.3%) and primary bloodstream infection (11.9%). The *Acinetobacter baumannii* (23.1%), *Escherichia coli* (10.9%) and *Pseudomonas aeruginosa* (9.7%) were the primary pathogens. Gram-negative infections, Gram-positive infections and mixed infections accounted for 33.5%, 9.9% and 11.7% of septic patients, respectively. The 28-day mortality rate in this study was 25.3%.

**Table 1:** Clinical characteristics of sepsis patients and healthy controls.

Variable	Sepsis (n = 403) N(%)	Control (n = 400) N(%)	P value
**Demographics**			
Age, years, mean ± SD	59.09 ± 16.66	57.47 ± 13.71	0.110
Male/female, number	286/117	271/129	0.323
**Sepsis status, n(%)**			
Mild sepsis	74(18.4)	N.A	
Severe sepsis	191(47.4)	N.A	
Septic shock	138(34.2)	N.A	
**Source of infection, n(%)**			
Respiratory tract infection	254(63.0)	N.A	
Primary bloodstream infection	48(11.9)	N.A	
Abdominal infection	94(23.3)	N.A	
Urinary tract infection	22(5.5)	N.A	
Catheter-associated infection	14(3.5)	N.A	
Brain	29(7.2)	N.A	
Others	35(8.7)	N.A	
**Infection types, n(%)**			
Gram-positive	40(9.9)	N.A	
Gram-negative	135(33.5)	N.A	
Mixed Gram-negative and -positive	47(11.7)	N.A	
Fungus	87(21.6)	N.A	
Polymicrobial	64(15.9)	N.A	
Negative blood culture	31(7.7)	N.A	
**Pathogenic bacteria, n(%)**			
Acinetobacter baumannii	93(23.1)	N.A	
Monilia albican	27(6.7)	N.A	
Yeast sample sporphyte	26(6.5)	N.A	
Aspergillus	17(4.2)	N.A	
Klebsiella pneumoniae	26(6.5)	N.A	
Pseudomonas aeruginosa	39(9.7)	N.A	
Staphylococcus aureus	33(8.2)	N.A	
Escherichia coli	44(10.9)	N.A	
Others	76(18.9)	N.A	
APACHE II score	23.8 ± 7.1	N.A	
28-day mortality, n(%)	102(25.3)	N.A	

N.A: not applicable; APACHE II: Acute Physiology and Chronic Health Evaluation II; Continuous data are expressed as the mean ± SD.

### The effect of ATG5 and ATG16L1 polymorphisms on the progression of sepsis

**Figure 1:**
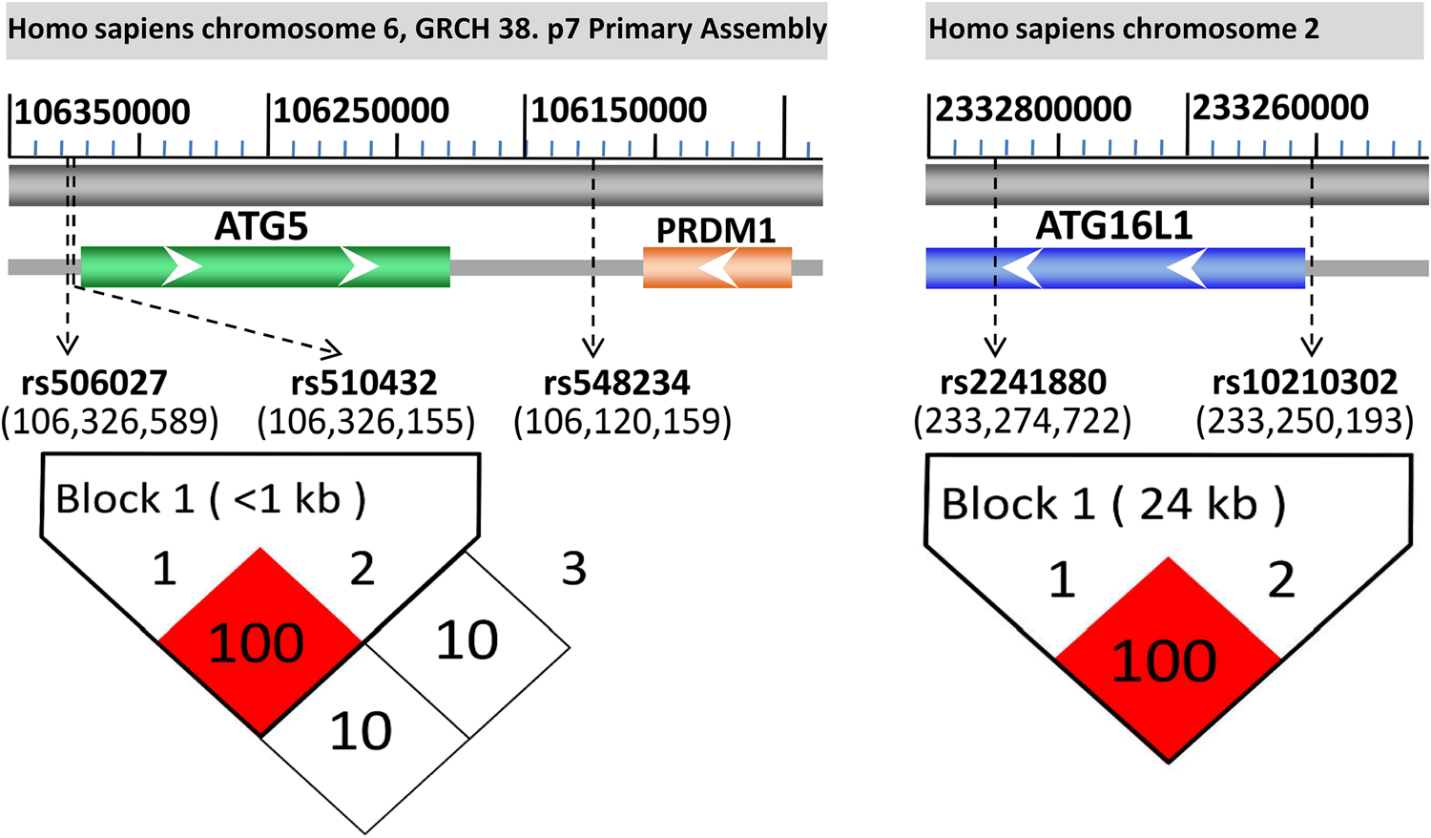
The linkage disequilibrium (LD) blocks (ATG5 and ATG16L1 genetic polymorphisms) and their locations in the genes. According to the GRCh38.p7 primary assembly, the human ATG5 and ATG16L1 genes are respectively located in Homo sapiens chromosome 6 (106,184,476–106,325,820) and Homo sapiens chromosome 2 (233,251,571–233,295,674). The rs506027 and rs510432 promoter polymorphisms are located in the upstream of the transcriptional start site (−769 bp and −335 bp), respectively. The rs548234 is located in the PRDM1-ATG5 intergenic region, and another promoter polymorphism of ATG16L1 rs10210302 is located in the upstream of the transcriptional start site (−1378 bp). Two haplotype blocks (rs506027–rs510432, D’ value = 100; rs10210302–rs2241880, D’ value = 100) are generated using Haploview 4.2.

All samples were sequenced and genotyped successfully and no deviations from Hardy-Weinberge quilibrium were observed for these ATG5 and ATG16L1 polymorphisms in sepsis and control groups (all P > 0.05, data not shown). We constructed two haplotype blocks (ATG5: rs506027–rs510432; ATG16L1: rs10210302–rs2241880) using the Haploview software program and analyzed the linkage disequilibrium (LD) of these polymorphisms. As presented in the Fig. 1, the ATG5 polymorphisms (rs506027, rs510432) and ATG16L1 polymorphisms (rs10210302, rs2241880) were both in complete linkage disequilibrium.

To evaluate whether three ATG5 polymorphisms (rs510432, rs506027 and rs548234) and two ATG16L1 polymorphisms (rs10210302 and rs2241880) were associated with sepsis progression, we separated the cases into mild sepsis, severe sepsis and septic shock subgroups on the basis of sepsis severity. As shown in Table 2, the genotype frequencies of the ATG5 promoter polymorphisms (rs510432 and rs506027) in the mild sepsis subgroup significantly differed from those in the severe sepsis subgroup (P = 0.013) and in the septic shock subgroup (P = 0.043). The ATG5 rs510432 A and rs506027 C allele frequencies in the subgroups of severe sepsis/septic shock were significantly higher than that in the subgroup of mild sepsis, which suggested that rs510432 (−335 G > A) and rs506027 (−769 T > C) within the promoter of ATG5 gene influenced patients predisposition to the progression of sepsis from mild sepsis to severe sepsis/septic shock. With regards to the genotype/allele frequencies of the other three polymorphisms (rs548234, rs10210302 and rs2241880), no significant differences were found among these three subgroups of sepsis.

**Table 2:** Genotype and allele frequencies distribution in the different sepsis status.

ATG SNP	Mild sepsis (n = 74)	Severe sepsis (n = 191)	Septic shock (n = 138)	Severe sepsis/Septic shock (n = 329)	P1	P2	P3	P1*	P2*	P3*
**rs510432/rs506027**										
GG/TT	34(45.9)	57(29.8)	44(31.9)	101(30.7)	0.013	0.043	0.012	0.026	0.043	0.020
GA + AA/TC + CC	40(54.1)	134(70.2)	94(68.1)	228(69.3)	—	—	—	—	—	—
G/T	100(67.6)	222(58.1)	154(55.8)	376(57.1)	0.046	0.018	0.020	0.046	0.036	0.020
A/C	48(32.4)	160(41.9)	122(44.2)	282(42.9)	—	—	—	—	—	—
**rs548234**										
TT	45(60.8)	96(50.3)	69(50.0)	165(50.2)	0.123	0.132	0.097	0.123	0.132	0.097
CT + CC	29(39.2)	95(49.7)	69(50.0)	164(49.8)	—	—	—	—	—	—
T	116(78.4)	272(71.2)	196(71.0)	468(71.1)	0.094	0.101	0.074	0.123	0.132	0.097
C	32(21.6)	110(28.8)	80(29.0)	190(28.9)	—	—	-	—	—	—
**rs10210302/rs2241880**										
CC/AA	31(41.9)	84(44.0)	67(48.6)	151(45.9)	0.758	0.354	0.532	0.758	0.354	0.532
CT + TT/GA + GG	43(58.1)	107(56.0)	71(51.4)	178(54.1)	—	—	—	—	—	—
C/A	94(63.5)	249(65.2)	194(70.3)	443(67.3)	0.718	0.154	0.374	0.758	0.308	0.532
T/G	54(36.5)	133(34.8)	82(29.7)	215(32.7)	—	—	—	—	—	—

P1: mild sepsis vs. severe sepsis; P2: mild sepsis vs. septic shock. *False discovery rate-adjusted P-value for multiple hypotheses testing using the Benjamin-Hochberg method.

### The effect of ATG5 and ATG16L1 genetic polymorphisms on the mortality of sepsis patients

**Figure 2:**
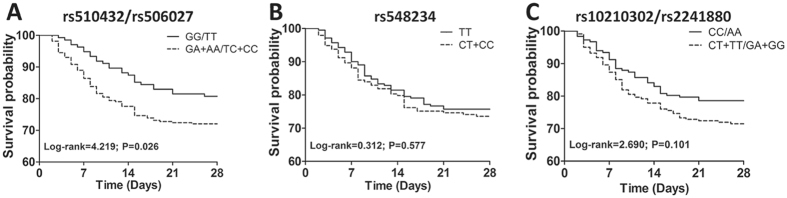
Kaplan-Meier survival analysis in sepsis patients. Analysis for effects of ATG5 rs510432/rs506027 (**A**) and rs548234 (**B**) polymorphisms and ATG16L1 rs10210302/rs2241880 (**C**) polymorphisms on survival of sepsis patients.

The genotype/allele frequency distributions of ATG5 and ATG16L1 polymorphisms in two subgroups stratified by 28-day mortality of sepsis patients were further evaluated. As shown in Table 3, comparing the genotype distributions between 28-day surviving and non-surviving patients, a tendency was observed towards higher frequencies of the rs510432 GA/AA and rs506027 TC/CC genotypes (Both P = 0.047) in non-surviving sepsis patients. Nonetheless, this difference showed no statistical significance after the Benjamini-Hochberg multiple-testing corrections (rs510432: GA/AA vs. GG *P = 0.118; rs506027: TC/CC vs. TT *P = 0.118). Furthermore, Kaplan-Meier survival analysis showed that rs510432 GA/AA and rs506027 TC/CC genotypes were significantly associated with the prognosis of sepsis patients. The mortality of sepsis patients carrying rs510432 GA/AA or rs506027 TC/CC genotypes increased with the extended length of stay in ICU gradually, and the 28-day survival exhibited much worse than in sepsis patients carrying rs510432 GG or rs506027 TT genotype in the end (Both log-rank = 4.219, P = 0.026; Fig. 2A). However, no significant differences were found concerning the other polymorphisms (rs548234: log-rank = 0.312, P = 0.577; rs10210302/rs2241880: log-rank = 2.690, P = 0.101; Fig. 2B,C).

**Table 3:** Genotype and allele frequencies distribution between 28-day surviving and non‐surviving sepsis patients.

SNP	Survivors n = 301(%)	Non-survivors n = 102(%)	P	P*	OR (95% CI)
**rs510432/rs506027**					
GG/TT	109(36.2)	26(25.5)	0.125	0.167	—
GA/TC	149(49.5)	57(55.9)	—	—	—
AA/CC	43(14.3)	19(18.6)	—	—	—
GG + GA/TT + TC	258(85.7)	83(81.4)	0.294	0.294	1.373(0.758, 2.488)
GA + AA/TC + CC	192(63.8)	76(74.5)	0.047	0.118	0.603(0.364, 0.997)
G/T	367(61.0)	109(53.4)			1.000 (reference)
A/C	235(39.0)	95(46.6)	0.059	0.118	0.735(0.533, 1.012)
**rs548234**					
TT	159(52.8)	51(50.0)	0.731	0.731	—
TC	122(40.5)	42(41.2)	—	—	—
CC	20(6.7)	9(8.8)	—	—	—
TT + CT	281(93.4)	93(91.2)	0.462	0.731	1.360(0.598, 3.091)
CT + CC	142(47.2)	51(50.0)	0.622	0.731	0.893(0.570, 1.400)
T	440(73.1)	144(70.6)	—	—	
C	162(26.9)	60(29.4)	0.490	0.731	0.884(0.622, 1.255)
**rs10210302/rs2241880**					
CC/AA	143(47.5)	39(38.2)	0.258	0.344	—
CT/GA	123(40.9)	50(49.0)	—	—	—
TT/GG	35(11.6)	13(12.8)	—	—	—
CC + CT/AA + GA	266(88.4)	89(87.3)	0.763	0.763	0.110(0.562, 2.192)
CT + TT/GA + GG	158(52.5)	63(61.8)	0.104	0.348	0.684(0.432, 1.082)
C/A	409(67.9)	128(62.7)	—	—	1.000 (reference)
T/G	193(32.1)	76(37.3)	0.174	0.348	0.795(0.571, 1.107)

OR: odds ratio; 95% CI: 95% confidence interval; *False discovery rate-adjusted P-value for multiple hypotheses testing using the Benjamin-Hochberg method.

### The distributions of the genotype/allele frequency between the sepsis and healthy controls

We then explored the possible associations of these genetic polymorphisms of ATG5 and ATG16L1 with the occurrence of sepsis. No statistically significant differences in the overall genotype/allele distributions of these five polymorphisms were found between the sepsis and control groups (all P > 0.05, Table 4), suggesting that these ATG5 and ATG16L1 genetic polymorphisms may not be associated with predisposition to sepsis.

**Table 4:** Frequencies of the ATG5 and ATG16L1 genotypes/alleles in the sepsis patients and controls.

SNP	Sepsis n = 403(%)	Control n = 400(%)	P	P*	OR (95% CI)
**rs510432/rs506027**					
GG/TT	135(33.5)	75(18.8)	0.239	0.239	—
GA/TC	206(51.1)	210(52.5)	—	—	—
AA/CC	62(15.4)	115(28.7)	—	—	—
GG + GA/TT + TC	341(84.6)	325(81.3)	0.205	0.239	1.269(0.877, 1.836)
GA + AA/TC + CC	268(66.5)	285(71.3)	0.146	0.239	0.801(0.594, 1.081)
G/T	476(59.1)	440(55.0)	—	—	1.000 (reference)
A/C	330(40.9)	360(45.0)	0.101	0.239	0.847(0.695, 1.033)
**rs548234**					
TT	210(52.1)	200(50.0)	0.707	0.752	—
CT	164(40.7)	174(43.5)	—	—	—
CC	29(7.2)	26(6.5)	—	—	—
TT + CT	374(92.8)	374(93.5)	0.696	0.752	0.897(0.518, 1.552)
CT + CC	193(47.9)	200(50.0)	0.550	0.752	0.919(0.697, 1.212)
T	584(72.5)	574(71.8)	—	—	1.000 (reference)
C	222(27.5)	226(28.2)	0.752	0.752	0.966(0.776, 1.201)
**rs10210302/rs2241880**					
CC/AA	182(45.2)	190(47.5)	0.800	0.800	—
CT/GA	173(42.9)	165(41.3)	—	—	—
TT/GG	48(11.9)	45(11.2)	—	—	—
CC + CT/AA + GA	355(88.1)	355(88.8)	0.770	0.800	0.938(0.608, 1.445)
CT + TT/GA + GG	221(54.8)	210(52.5)	0.506	0.800	1.099(0.832, 1.450)
C/A	537(66.6)	545(68.1)	—	—	1.000 (reference)
T/G	269(33.4)	255(31.9)	0.522	0.800	1.071(0.869, 1.319)

OR: odds ratio; 95% CI: 95% confidence interval; *False discovery rate-adjusted P-value for multiple hypotheses testing using the Benjamin-Hochberg method.

### The effect of ATG5 and ATG16L1 polymorphisms on the expression of ATG5 and ATG16L1

**Figure 3:**
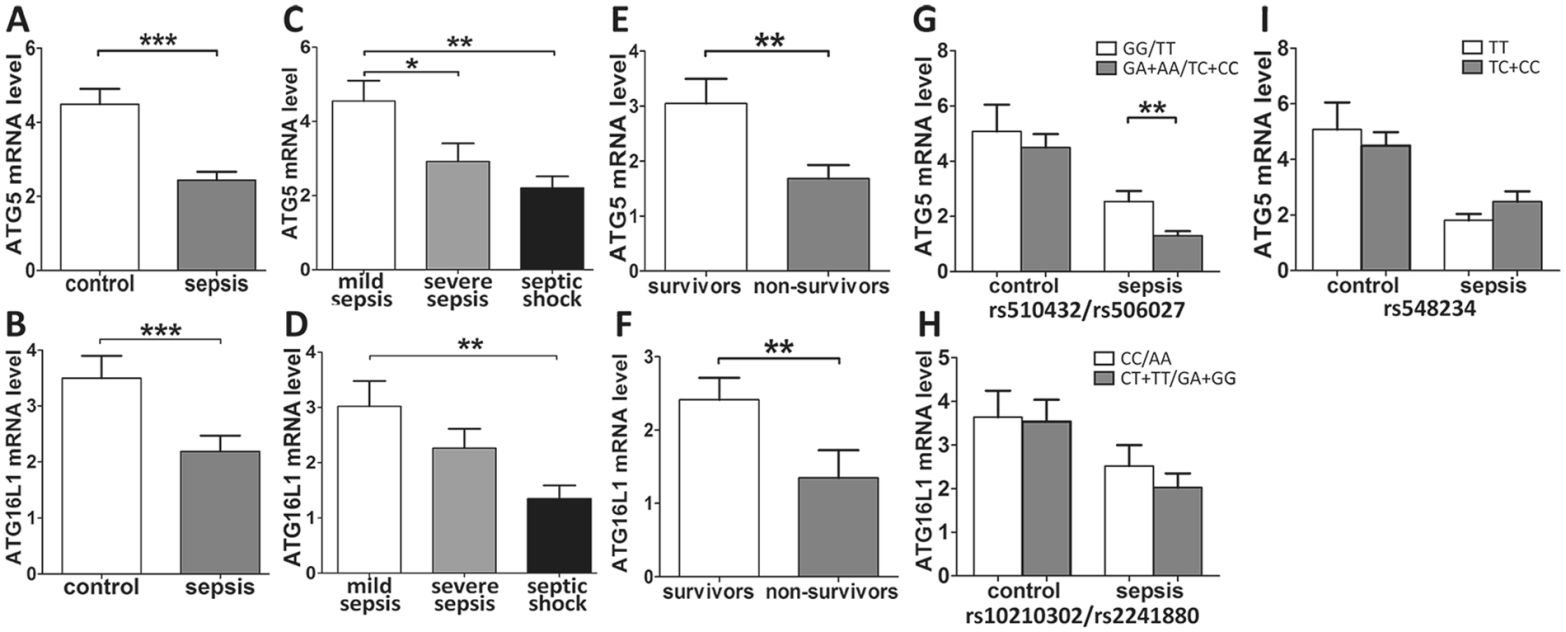
Real-time PCR analysis of the ATG5 and ATG16L1 mRNA expression in sepsis patients and healthy controls. Expression levels of ATG5 and ATG16L1 in sepsis patients and healthy controls (**A**,**B**). Expression levels of ATG5 and ATG16L1 in mild sepsis, severe sepsis and septic shock subgroups (**C**,**D**). Expression levels of ATG5 and ATG16L1 in 28-day surviving and non-surviving sepsis patients (**E**,**F**). The distribution of ATG5 expression in groups of sepsis patients with different rs510432/rs506027 genotypes (**G**) and different rs548234 genotypes (**I**). The distribution of ATG16L1 expression in groups of sepsis patients with different rs10210302/rs2241880 genotypes (**H**). The horizontal line represents the mean expression levels of ATG5 and ATG16L1 with each group. *P < 0.05; **P < 0.01; ***P < 0.001.

A comparative analysis was performed with respect to the mRNA expression of ATG5 and ATG16L1 in the PBMCs of 80 sepsis cases and 80 controls. Our results showed that the ATG5 and ATG16L1 expression levels in the sepsis cases were significantly reduced relative to the controls (P < 0.001; Fig. 3A,B), and it is worth nothing that the ATG5 and ATG16L1 mRNA expression levels decreased significantly with the aggravation of sepsis (P < 0.05; Fig. 3C,D). The expression levels of ATG5 and ATG16L1 in the 28-day non-surviving patients with sepsis were significantly decreased compared to the 28-day surviving patients (P < 0.01; Fig. 3E,F). The sepsis patients bearing the ATG5 rs510432 GA/AA and rs506027 TC/CC genotypes exhibited significantly lower expression of ATG5 compared to the rs510432 GG and rs506027 TT genotype carriers (Both P = 0.009; Fig. 3G). No significant differences in ATG16L1 expression levels were found for the different ATG16L1 polymorphisms in the sepsis or control groups (Fig. 3H).

**Figure 4:**
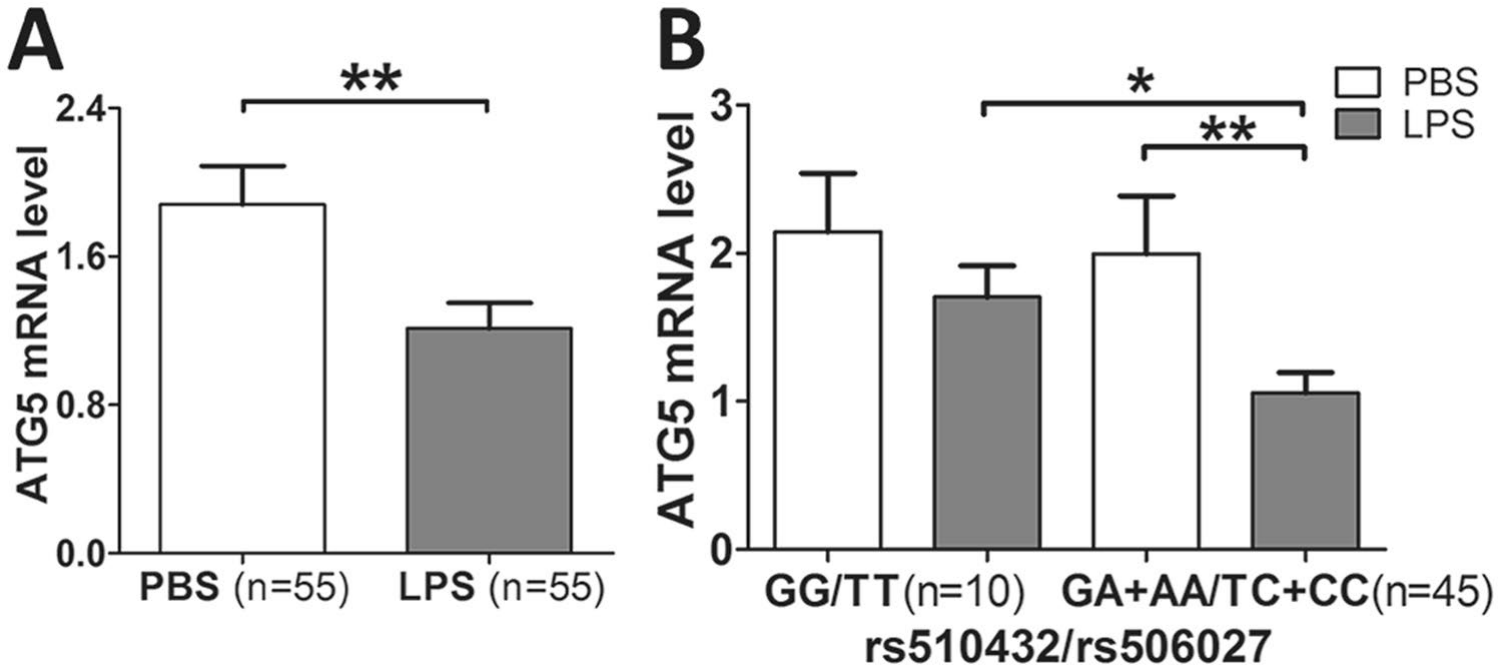
Real-time PCR analysis of the ATG5 expression in the mononuclear cells isolated from 55 healthy volunteers. The ATG5 expression in PBMCs of 55 healthy individuals under LPS stimulation (500 ng/mL) (**A**). The ATG5 expression with different genotypes of ATG5 rs510432/rs506027 polymorphisms in PBMCs from 55 healthy individuals under LPS stimulation (**B**). The horizontal line indicates the mean expression level with each genotype groups. *p < 0.05; **p < 0.01; ***p < 0.001.

**Figure 5:**
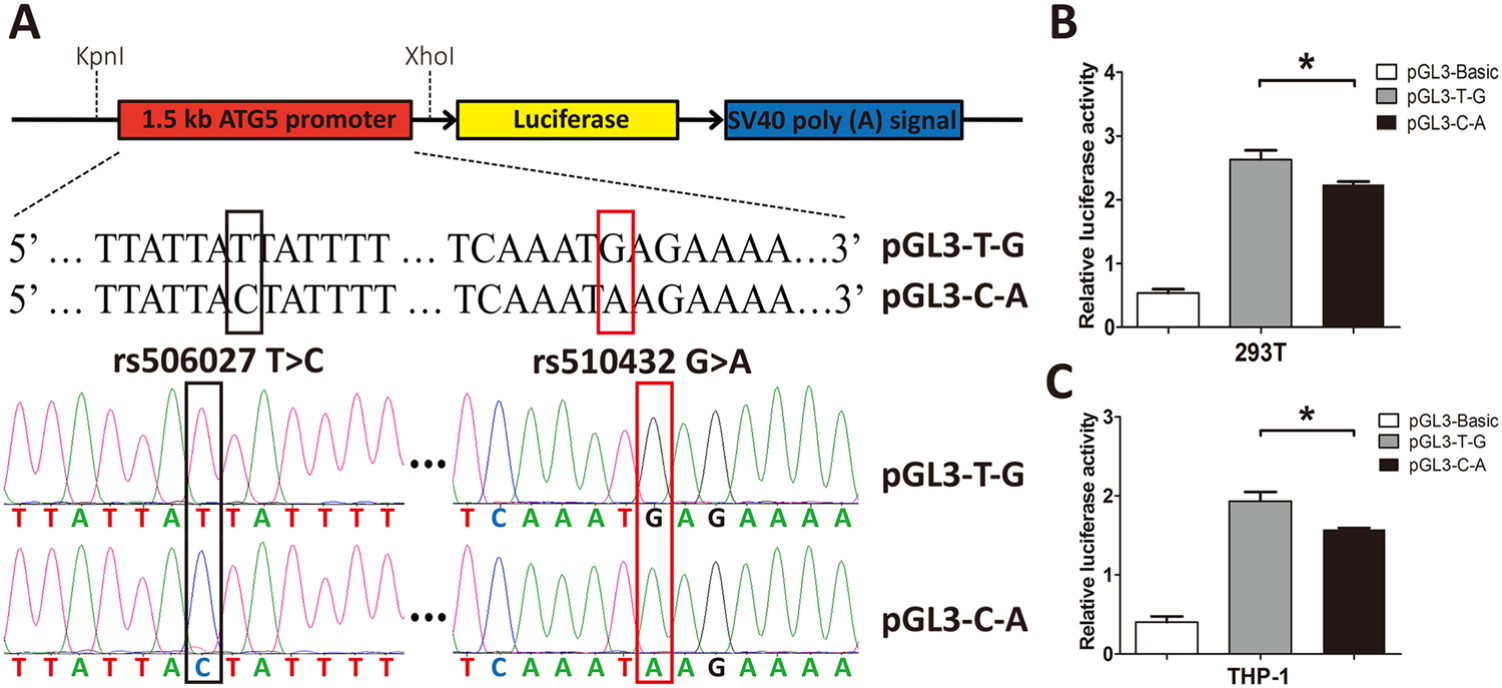
The constructs of ATG5 plasmid and functional promoter activities of ATG5 polymorphisms in 293 T and THP-1 cells. The promoter region of ATG5 DNA sequence (1500 bp; Homo sapiens chromosome 6: 106,325,800–106,327,300) carrying T-G or C-A of rs506027 T > C and rs510432 G > A were cloned into pGL3 luciferase reporter vectors (**A**). The 293 T and THP-1cells were transfected with the ATG5 plasmid constructs for 48 h, and the promoter activities were detected by dual-luciferase report assays (**B**,**C**). Three parallel samples were used in all transfections, and all experiments were performed in triplicate. *p < 0.05; **p < 0.01; ***p < 0.001.

To further confirm the effect of ATG5 promoter polymorphisms (rs510432 and rs506027) on the gene expression of ATG5 *in vitro*, we stimulated the mononuclear cells isolated from 55 healthy volunteers bearing different ATG5 rs510432 and 506027 genotypes with LPS for 8 hours. The expression level of ATG5 was observed to be significantly decreased in mononuclear cells after LPS stimulation (P = 0.008; Fig. 4A). Importantly, the ATG5 expression was significantly decreased after LPS stimulation in mononuclear cells from the individuals carrying the ATG5 rs510432 GA/AA or rs506027 TC/CC genotypes (n = 45) (Fig. 4B). Nevertheless, the mononuclear cells isolated from individuals who were bearing the rs510432 GG or rs506027 TT genotypes (n = 10) showed no statistical differences in ATG5 expression after the stimulation (Fig. 4B). Furthermore, the luciferase assays were performed using the 293 T and THP-1 cell lines to characterize the effects of the rs506027–rs510432 ATG5 promoter haplotypes on gene transcription. As shown in Fig. 5, the rs506027–rs510432 T-G haplotype in the ATG5 promoter exhibited significantly higher promoter activities compared to the C-A haplotype mutation in 293 T and THP-1 cells.

### Effects of ATG5/ATG16L1 polymorphisms on the expression of related inflammatory cytokines

**Figure 6:**
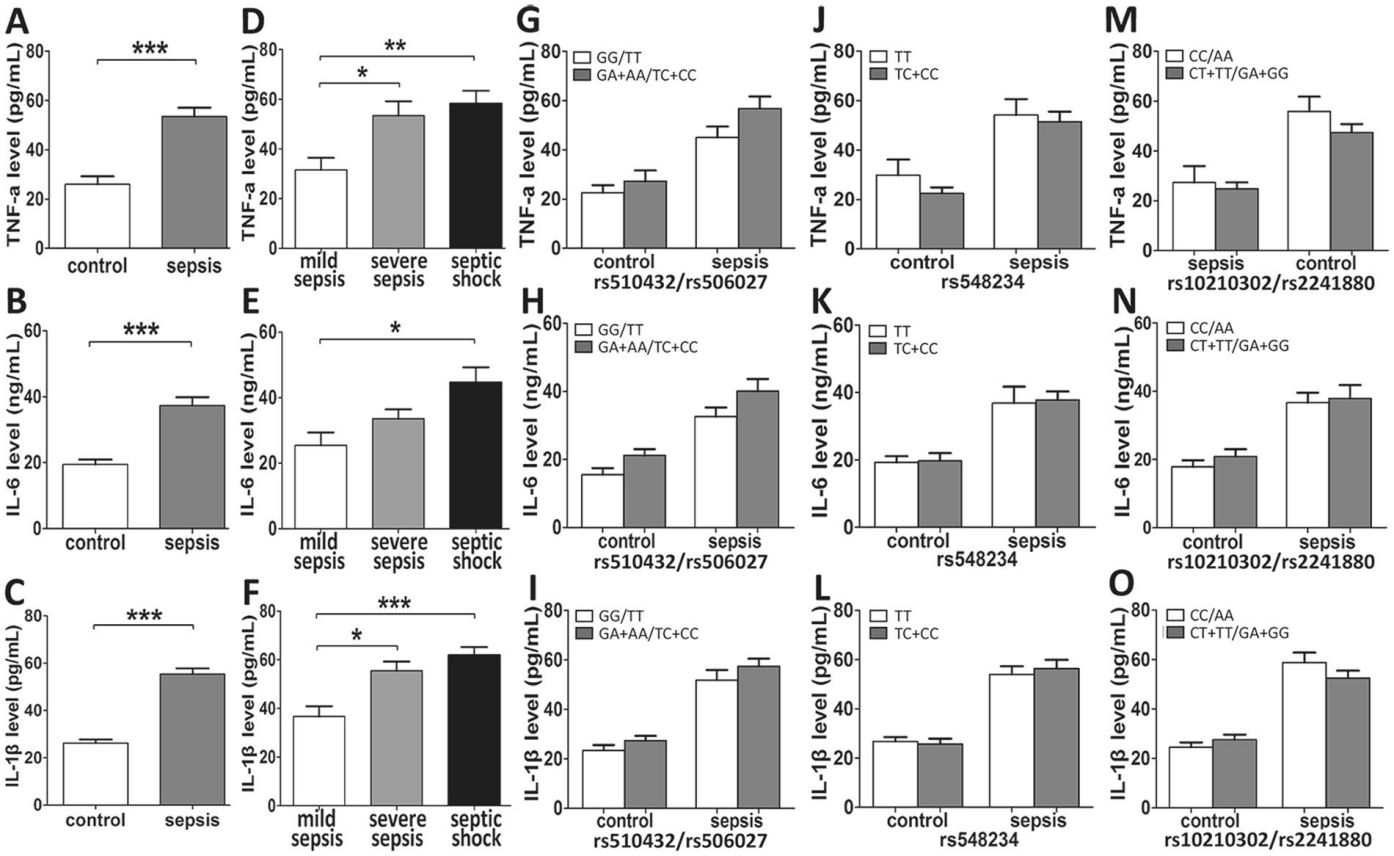
The concentrations of related inflammatory cytokines in sepsis patients and healthy controls. The concentrations of TNF-α (**A**), IL-6 (**B**) and IL-1β (**C**) in sepsis patients (n = 80) and healthy controls (n = 80). The concentrations of TNF-α (**D**), IL-6 (**E**) and IL-1β (**F**) in mild sepsis, severe sepsis and septic shock subgroups. The genotype distribution between the ATG5 polymorphisms and the concentrations of TNF-α, IL-6 and IL-1β (**G**–**L**). The genotype distribution between the ATG16L1 polymorphisms and the concentrations of TNF-α (**M**), IL-6 (**N**) and IL-1β (**O**). The horizontal line represents the mean concentrations of the related cytokines with each group. *P < 0.05; **P < 0.01; ***P < 0.001.

Based on the effect of the ATG5 rs510432 and rs506027 polymorphisms on the ATG5 expression, we further evaluated the potential associations of these genetic polymorphisms with the expression of related inflammatory cytokines. The plasma concentrations of TNF-α, IL-6 and IL-1β were significantly higher in the sepsis patients compared to the healthy volunteers (P < 0.0001; Fig. 6A,B,C), and that they increased with the aggravation of sepsis (P < 0.05; Fig. 6D,E,F). However, no significant differences in the TNF-α, IL-6 or IL-1β plasma concentrations were found among these five different polymorphisms (rs510432, rs506027, rs548234, rs10210302 and rs2241880) (all P > 0.05; Fig. 6).

**Figure 7:**
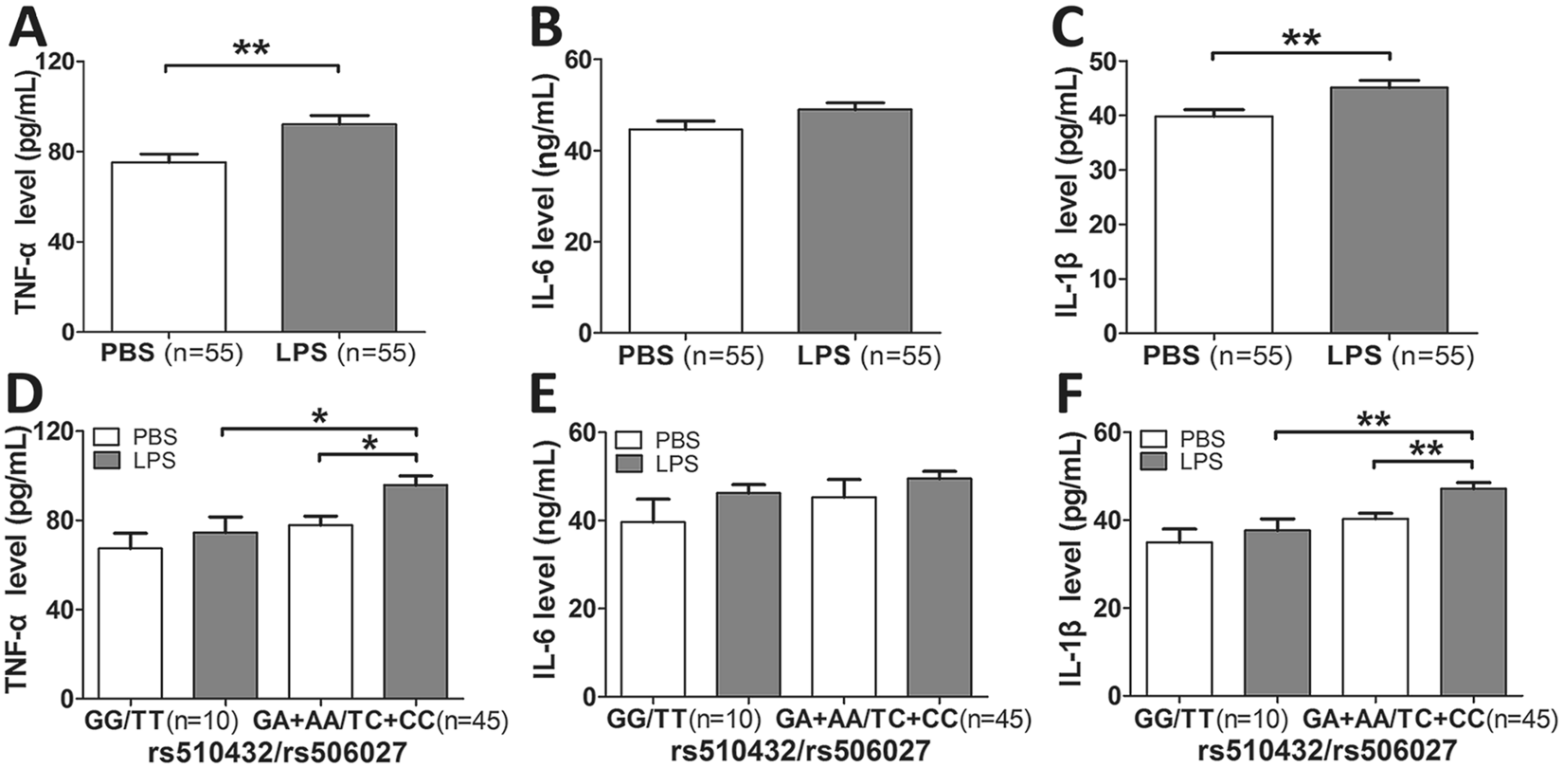
Polymorphisms of ATG5 enhanced the inflammatory cytokines release of human monocytes. PBMCs isolated from 55 healthy volunteers carrying different genotypes of ATG5 rs510432/rs506027 polymorphisms were incubated in RPMI1640 (10% fetal calf serum) and stimulated with 500 ng/mL LPS for 8 h. TNF-α, IL-6 and IL-1β were measured in the supernatants by ELISA (**A–F**). The horizontal line indicates the mean levels with each genotype groups. *p < 0.05; **p < 0.01; ***p < 0.001.

Another comparative determination was conducted to ascertain the genetic influence of these polymorphisms on the pro-inflammatory cytokines production (TNF-α, IL-6 and IL-1β) in the mononuclear cells isolated from 55 healthy volunteers bearing different ATG5 rs510432 and 506027 genotypes under LPS stimulation *in vitro*. As presented in the Fig. 7, no significant differences of these inflammatory cytokines production were observed among the different genotypes of ATG5 polymorphisms without LPS stimulation. However, TNF-α and IL-1β production was significantly higher in mononuclear cells isolated from the individuals carrying ATG5 rs510432 GA/AA or rs506027 TC/CC genotypes (n = 45) under LPS stimulation, compared to rs510432 GG or rs506027 TT genotype carriers (n = 10).

### Knockdown of ATG5 enhances inflammatory cytokines release in response to LPS stimulation

**Figure 8:**
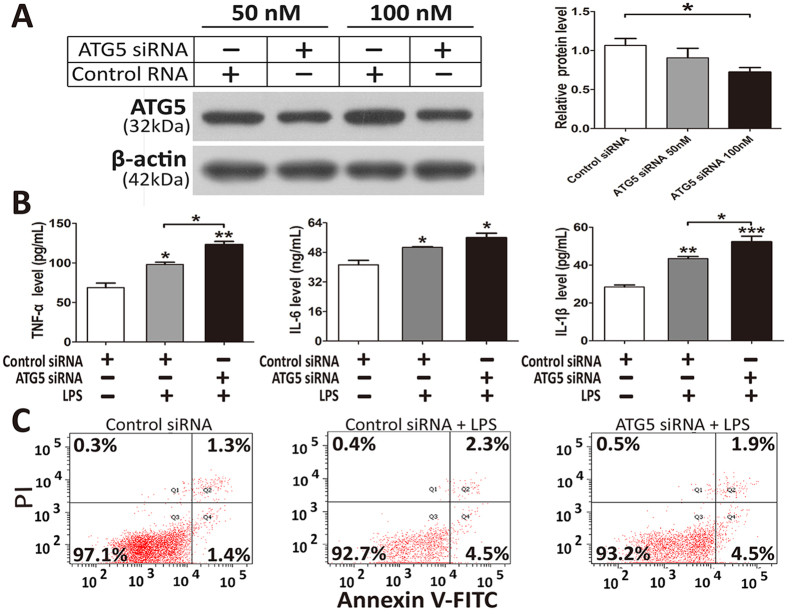
Inhibition of autophagy by ATG5 siRNA treatment elevated secretion of inflammatory cytokines in THP-1 cells. Western blotting of ATG5 in THP-1 cells transfected with ATG5 siRNA or control siRNA for 72 h (**A**). Full-length blots are presented in Supplementary Fig. S1; Supernatants from THP-1 cells treated with 500 ng/mL LPS for 8 h after transfection with ATG5 siRNA or control siRNA for 72 h were collected and ELISA was performed for TNF-α, IL-6 and IL-1β (**B**). Evaluation of apoptosis of THP-1 cells treated with 500 ng/mL LPS for 8 h after transfection with ATG5 siRNA or control siRNA for 72 h by using annexin-V-FITC/PI staining and flow cytometry (**C**). Bar graphs indicate the mean ± SEM for a minimum of three experiments, each performed in triplicate. *p < 0.05; **p < 0.01; ***p < 0.001.

We next evaluated the effect of autophagy protein ATG5 on the cellular apoptosis and secretion levels of TNF-α, IL-6 and IL-1β in THP-1 cells in response to LPS stimulation. As presented in Fig. 8A, transfection with ATG5-specific siRNA led to an effective knockdown of ATG5 protein expression in THP-1 cells. Compared with THP-1 cells transfected with scrambled control siRNA, the cells transfected with ATG5 siRNA showed higher expression levels of TNF-α and IL-1β under LPS stimulation (Fig. 8B). In addition, THP-1 cells stimulated with LPS exhibited a 4.1% increase in apoptotic and necrotic cell death as measured by the ANXA5/Annexin V-FITC apoptosis detection (Fig. 8C). However, no significant differences in the apoptotic and necrotic cell death were observed between ATG5 siRNA-treated and control siRNA-treated cells under LPS stimulation.

## Discussion

In this hospital-based case-control study, we investigate the clinical relevance of three ATG5 polymorphisms (rs510432, rs506027 and rs548234) and two ATG16L1 polymorphisms (rs10210302 and rs2241880) for susceptibility to and progression of sepsis in a Chinese Han population. ATG5, an essential component of the autophagosome, interacts with ATG16L1 and ATG12 in the formation of the ATG12-ATG5-ATG16L1 complex, which plays a significant role in the initiation process of autophagy^35, 36^. Increasing evidence has recently indicated that sepsis patients and animal models of sepsis exhibit reduced ATG5/ATG16L1 expression and autophagic activity in the late stage of sepsis and this lagging suppression of autophagic activity may contribute to inflammatory dysregulation, mitochondrial dysfunction and apoptosis, which are strongly correlated with organ dysfunction and mortality following sepsis^37–40^. It has also been reported that the autophagic dysfunction induced by genomic deletion of ATG5 or ATG16L1 has a lethal effect in murine models of sepsis^23–25^, whereas therapeutic activation of autophagy protects against septic insults^41, 42^. Our results showed that ATG5 and ATG16L1 expression levels in sepsis patients were significantly reduced relative to healthy controls, and that it decreased with the aggravation of sepsis which was consistent with these previous studies. Furthermore, the expression levels of ATG5 and ATG16L1 in the 28-day non-surviving patients with sepsis were significantly decreased compared to the 28-day surviving patients, suggesting significant roles of ATG5 and ATG16L1 in the progression and outcome of sepsis, which may have potentially important therapeutic implications of sepsis and other inflammation-related disease.

Multiple lines of evidence have demonstrated that several ATG5 polymorphisms influence patient predisposition to various inflammatory diseases, including asthma, SLE and Parkinson’s disease, by altering ATG5 expression levels and autophagy activity^26–28^. Among ATG16L1 genetic polymorphisms, rs10210302 and rs2241880 were implicated in patients’ predisposition to Paget bone disease, psoriasis and Crohn’s diseases^43–45^. And this present study indicated a significant association of ATG5 promoter polymorphisms with the progression of sepsis that the rs510432 A and rs506027 C alleles were both overrepresented among the severity of sepsis. The mortality of sepsis patients carrying the two genotypes also increased with the extended length of stay in ICU gradually, and the 28-day survival exhibited much worse than in sepsis patients carrying rs510432 GG or rs506027 TT genotype, further supporting that both rs510432 G > A and rs506027 T > C are statistically significant prognostic factors that may act as significant genetic indicators for predicting the sepsis progression. Nonetheless, no statistically significant associations were found between ATG16L1 polymorphisms and sepsis susceptibility or progression, which was inconsistent with the already reported study that the ATG16L1 rs2241880 A allele was associated with sepsis severity in sepsis patients of Caucasian origin^46^. This discrepancy may be due to the different clinical and biological parameters of diseases and the different ethnic origins of studied population, and this association needs to be further confirmed in a larger samples and different populations.

It has been demonstrated that the ATG5 rs506027–rs510432 T-G promoter haplotype was associated with higher promoter activity^27^, and ATG5 rs510432 G allele increased promoter activity relative to A allele^47^. Consistently, we conducted luciferase assays in 293 T and THP-1 cells to evaluate the effect of ATG5 rs506027–rs510432 promoter haplotypes on transcription efficiency and confirmed that the rs506027–rs510432 T-G haplotype in ATG5 gene promoter exhibited significantly higher promoter activities compared to the C-A haplotype mutation, suggesting that the sepsis-associated rs506027 and rs510432 polymorphisms played pivotal roles in regulating the gene transcription of ATG5 through the modulation of promoter activity. Furthermore, we tested *in vitro* stimulation study with LPS to confirm the effect of these two functional promoter polymorphisms on the ATG5 gene expression. Our results confirmed that ATG5 expression was significantly decreased in individuals carrying rs510432 GA/AA or rs506027 TC/CC genotypes under LPS stimulation. Interestingly, no significant differences in ATG5 expression were found among different genotypes of ATG5 polymorphisms without LPS stimulation, suggesting that these two SNPs may play the role in the case of stress, which also provided evidence that the ATG5 promoter polymorphisms influenced the sepsis progression rather than the occurrence of sepsis.

The overwhelming production of pro-inflammatory cytokines is considered the primary culprit of sepsis, resulting in tissue injury and functional disruption of multiple organs^48, 49^. Autophagy is involved in several pro-inflammatory signaling pathways following sepsis^50, 51^. A recent study demonstrated that autophagy in macrophages modulates the clearance of infectious pathogens and the inflammasome-dependent production of pro-inflammatory cytokines^52^. Genetic deletion of ATG5 or ATG16L1 increases the secretion levels of IL-1β, TNF-α and IL-6 in macrophages and has lethal effects on mice following sepsis^21–24^. Therefore, we evaluated the effect of autophagy protein ATG5 on the cellular apoptosis and secretion levels of these related cytokines in THP-1 cells in response to LPS stimulation. It has been reported that the autophagy dysfunction induced by ATG5 knockdown may promote accumulation of dysfunctional mitochondria, active inflammasomes and cytosolic translocation of mitochondrial DNA in response to LPS in monocyte-macrophages^22, 52–54^, which ultimately results in increased production of pro-inflammatory cytokines and exaggerated inflammatory responses. As expected, our results showed that the ATG5 knockdown led to significantly higher expression levels of TNF-α and IL-1β in the THP-1 cells under the stimulation of LPS, which was consistent with these previous studies. Given the evidence implicating significant roles of the autophagy protein ATG5 in the inflammatory response during sepsis, we further evaluated the possible associations between these genetic polymorphisms and the production of these related cytokines in sepsis patients. However, no significant differences in the plasma concentrations of these related cytokines were observed for different genotypes of these five examined genetic polymorphisms of ATG5 and ATG16L1 in sepsis patients. We inferred that these results may be attributed to the vast and complex inflammatory system in the whole body’s environment by which the genetic effects of the functional polymorphisms may not be adequately powerful enough to significantly affect the inflammatory signaling during sepsis. Notably, the results of the LPS stimulation study *in vitro* indicated that the ATG5 rs510432 A or rs506027 C allele enhanced the release of TNF-α and IL-1β in the mononuclear cells isolated from individuals in response to LPS stimulation. Future studies will evaluate the molecular mechanisms involved in these two functional polymorphisms by using a promoter prediction technique in concert with experimental verification in cellular and mouse models of sepsis.

Several limitations should be acknowledged in this study. First, the sample number in this study was relatively limited, and the subjects enrolled in this study were only from the Han Chinese ethnic group. Therefore, further studies with a larger sample size of sepsis patients from different ethnic origins are needed to verify our preliminary conclusions. Second, in this study only five ATG5 and ATG16L1 genetic polymorphisms implicated in the susceptibility and progression of sepsis were examined, whereas other polymorphisms of ATG5/ATG16L1 and other autophagy-related genes that may be associated with sepsis remained to be identified. Third, although we excluded septic patients with specific diseases to attain greater homogeneity in the samples, it remains possible that the ATG5 and ATG16L1 genotypes examined here have roles in diseases that were excluded from this study; our further study will focus on mechanism of the autophagy-related genes and functional polymorphisms in the progression of sepsis.

## Conclusions

In this present study, we provide evidence showing a significant association of ATG5 promoter polymorphisms with predisposition to sepsis progression in a Chinese Han population. The rs510432 GA/AA and rs506027 TC/CC genotypes variants also aggravated the 28-day mortality in sepsis patients. Functional assays suggested that the sepsis-associated risk alleles of rs510432 A - rs506027 C may decrease the gene transcription of ATG5 via modulating its promoter activity, then leading to the enhanced release of pro-inflammatory cytokines, ultimately contributing to the susceptibility to sepsis progression and poor prognosis.

## Methods

### Participant Recruitment

We enrolled 403 sepsis patients (aged 23–86; 286 men and 117 women) within 24 hours of admission to the intensive care unit (ICU) at the Affiliated Hospital of Guangdong Medical University (Zhanjiang, China) from December 2012 to April 2016, and 400 healthy normal volunteers (age 20–83; 271 men and 129 women) from the Health Examination Center in this hospital during the same time period. Patients who suffered from human immunodeficiency virus (HIV), cancer, blood diseases, autoimmune diseases or receiving immunosuppressive therapy were excluded from this study. The peripheral blood samples were collected from the enrolled sepsis patients within 12 hours when the diagnosis of sepsis, severe sepsis, or septic shock was established. The sepsis, severe sepsis or septic shock is the initial situation of the disease in the patients. The following clinical parameters were recorded for each patient: age, sex, dysfunctional organs, source of infection, blood microbiological cultures, and Acute Physiology and Chronic Health Evaluation (APACHE) II score^55^. Sepsis was defined on the basis of the International Sepsis Definitions Conference^33, 34^. *In vitro* lipopolysaccharide (LPS) stimulation test was conducted with peripheral blood mononuclear cells (PBMCs) isolated from a group of 55 healthy volunteers. The participants in the healthy control group were free from any history of sepsis, cancer, autoimmune diseases and other inflammation-related diseases. All the enrolled subjects were from the Chinese Han population and were at least of eighteen years old. This study was approved by the Ethical Committee of the Affiliated Hospital of Guangdong Medical University (No. PJ2012135), and written informed consent was obtained from all the healthy volunteers and patients. All experimental methods were carried out in accordance with the approved guidelines.

### Blood sampling, DNA extraction and genotyping

Three milliliters of the peripheral blood from the enrolled subjects were drawn in EDTA-containing tubes; 1 ml were collected for the DNA extraction and the isolation of PBMCs. Another 2 ml in the tubes were centrifuged at low speed and the plasma was stored at −80 °C until processed. The IANamp Blood DNA Kit (Tiangen Biotech Co., Ltd., Beijing, China) was used for the extraction of the genomic DNA following the instructions of the manufacturer. Five genetic polymorphisms of ATG5 (rs510432, rs506027, rs548234) and ATG16L1 (rs10210302, rs2241880) were genotyped by using the SNaPshot Multiplex Kit (Applied Biosystems Co., Ltd., Foster City, CA, USA). The primers used for PCR amplification and extension of SNaPshot were as follows: rs506027F, 5′ CGGTGGCGGGCTTCTGTAGT 3′; rs506027R, 5′ CATGGCGTTAAGGCACGTGTAA 3′; rs510432F, 5′ TCCCTTTCTTCCTGGCATCACA 3′; rs510432R, 5′ GCCCGGCCCCAATTATTTTTAC 3′; rs548234F, 5′ GCTTTCTGGAAATTAGCTGGGCTCT 3′; rs548234R, 5′ GGAACCTCAATCTCTTGCGCTCT 3′; rs10210302F, 5′ CCTTCCAACGACTTCAGGTCCA 3′; rs10210302R, 5′ GCATGAAAATGGAGCCAG-ACAA 3′; rs2241880F, 5′ CGCTCTGTCTCTTCCTTCCCAGT 3′; rs2241880R, 5′ CGGGGCTGAAGCATAC-TTACGA 3′; The SNaPshot PCR reaction (10 μL) contained 5 μL of SNaPshot Multiplex Kit reagent (ABI), 4 μL of templates and 1 μl of primer mix. The PCR reaction protocol was as follows: 96 °C for 60 s; 28 cycles of 96 °C for 10 s, 55 °C for 5 s, and 60 °C for 30 s; 4 °C for 120 s. The purified products (0.5 μL) mixed with Lizl20 Size Standard (0.5 μL) and HiDi formamide (9 μL) were incubated at 95 °C for 5 minutes, and then were detected by ABI Prism 3730XL genetic sequence analyzer (Applied Biosystems, Foster City, CA, USA) and GeneMapper 4.1 (Applied Biosystems, Carlsbad, CA, USA). At last, 10% of the samples were randomly chosen as the validation group for re-genotyping. Power analyses performed using QUANTO 1.2 software exhibited 98.2% power for rs506027, 98.2% power for rs510432, 96.7% power for rs548234, 97.4% power for rs10210302 and 97.4% power for rs2241880 to test a genotype relative risk at an odds ratio of 1.5 at a significance level of 0.05 in this study.

### Plasma collection and mononuclear cells isolation

160 enrolled subjects (80 sepsis cases and 80 healthy volunteers) were randomly selected for the plasma collection and mononuclear cells isolation from their blood samples. The 80 sepsis samples included 12 mild sepsis, 38 severe sepsis and 30 septic shock samples. Among the 160 selected subjects, 29 patients and 20 healthy volunteers carried the rs510432 GG and rs506027 TT genotypes, 35 patients and 39 healthy volunteers carried the rs548234 TT genotype, 35 patients and 37 healthy volunteers carried the rs10210302 CC and rs2241880 AA genotypes. The plasma was extracted from the blood samples by centrifugation at low speed and stored at −80 °C until used for the measurement of TNF-α, IL-6 and IL-1β. The isolation of PBMCs was performed by density gradient centrifugation method with Lymphoprep^TM^ (Axis-Shield PoCAS, Oslo, Norway). Briefly, the peripheral blood was added with the same volume of 0.9% NaCl, and the diluted blood was transferred into a tube which consists of a Ficoll premium solution in order to layer the blood. The blood was then centrifuged at 800 × g for 30 minutes at room temperature to make the mononuclear cells form a distinct band at the medium interface. Then the mononuclear cells were collected into a new tube and washed with 0.9% NaCl, and then centrifuged at 250 × g for 10 minutes, stored at −80 °C until used.

### LPS stimulation experiments on the PBMCs

The PBMCs from the 55 healthy volunteers were obtained following the same procedures as the above. The PBMCs were washed twice in saline and then seeded in 24-well plates and cultured in RPMI 1640 medium (Thermo Fisher Scientific, Waltham, MA, USA) supplemented with 10% fetal bovine serum (FBS; Thermo Fisher Scientific) and 1% antibiotic mixture (penicillin/ streptomycin; HyClone, Logan, UT, USA) under a 5% CO_2_ atmosphere at 37 °C. After treatment with LPS (500 ng/mL) for 8 h, the PBMCs were harvested for the RNA extraction, and the supernatants were extracted and stored at −80 °C until used. Treatment with PBS was used for control cells.

### Cell culture

The human acute monocytic leukemia cell line (THP-1) and 293 T cells were obtained from Shanghai Institute of Cell Biology (Shanghai, China). The cells were incubated in RPMI 1640 medium supplemented with 1% antibiotic mixture and 10% fetal bovine serum under a 5% CO_2_ atmosphere at 37 °C.

### ATG5 plasmid constructs for promoter assays and luciferase assay

We cloned 1500 bp (Homo sapiens chromosome 6: 106,325,800–106,327,300, the promoter region of ATG5 carrying rs510432 G > A and rs506027 T > C) of DNA sequence located upstream of the transcription start site of the ATG5 gene into pGL3 luciferase reporter vectors (Promega, Madison, Wisconsin, USA): rs506027–rs510432-T-G and rs506027–rs510432-C-A. The ATG5 promoter gene fragments were amplified by PCR using the following primers: forward primer 5′ TCCACGCGTCATGTAATCCTCCTTCCTCAAC 3′ and reverse primer 5′ AATCTCGAGTTCCGCCCTCTG-GTATCCAG 3′. The 293 T and THP-1 cells were seeded onto 12-well plate with a concentration of 10 × 10^4^/well to obtain 70% to 80% confluence. Cells were then transfected with the luciferase pGL3 haplotype reporter, Renilla luciferase pGL3 vectors and Lipofectamine 2000 (Invitrogen, USA) following the manufacturer’s protocol. Three parallel samples were used in all transfections, and all experiments were performed in triplicate. The assay was conducted by using the dual-luciferase assay kit (Promega, USA), and the luminescence was measured using a Mithras LB940 Multilabel Reader (Berthold Technologies, Bad Wildbad, Germany).

### RNA interference and LPS stimulation

Small interfering RNA (siRNA) specific to ATG5 and control siRNA were synthesized by Shanghai GenePharma Biotechnology (Shanghai, China). The sense strand sequence of the ATG5 siRNA was as follows: 5′ AAUUCGUCCAAACCACACAUCUCGA 3′. THP-1 cells were transfected with either ATG5 siRNA or control siRNA using Lipofectamine2000 (Invitrogen, USA) following the manufacturer’s protocol. Briefly, the cells were seeded onto 12-well plate to obtain 70% to 80% confluence. Before transfection, the ATG5 siRNA (50 nM or 100 nM) was incubated with Lipofectamine 2000 in OPTI-MEM^TM^ reduced serum medium (Invitrogen, USA) for 20 minutes. The mixture of Lipofectamine 2000 and siRNA was added to individual wells containing the cells and growth medium with no antibiotic and incubated for 8 h at 37 °C. Then the culture medium was changed. After 72 h of transfection, western blotting was conducted to evaluate the efficiency of target gene silencing. After 72 h of transfection, the cells were treated with LPS (500 ng/mL) for 8 h. Transfection with control siRNA and treatment with PBS were used for control cells.

### RNA extraction and quantitative real-time PCR

The extraction of RNA from PBMCs was performed by using the RNAprep Pure Blood Kit (Sangon Biotech, Shanghai, China). The First Strand cDNA Synthesis Kit (Thermo Fisher Scientific, Waltham, MA, USA) was used to convert the RNA into cDNA according to the instructions of the manufacturer. The expression levels of ATG5 and ATG16L1 mRNA were detected by quantitative real-time PCR with the SYBR green method. The primers were designed with Primer Premier 5.0 software by Shanghai Sangon Biological Engineering as follows: ATG5: 5′ ATGAAGGCACACCACTGAAATG 3′ and 5′ GCATCCTTAGATGGACAGTGC 3′; ATG16L1: 5′ AGGACAGGGAGATGCAGATG 3′ and 5′ CTCGCCTCCAGCATGAAAGT 3′; ACTB (β-actin): 5′ TCCCTGGAGAAGAGCTACGA 3′ and 5′ AGCACTGTGTTGGCGTACAG 3′. The 10 μL final volume of the PCR amplification reaction consisted of SYBRII Green PCR master mix (5 μL, TaKaRa), each of specific forward and reverse primers (0.2 μL), ddH_2_O (3.6 μL), and cDNA (1 μL). The quantitative real-time PCR was conducted using a LightCycler480 sequence detector system (Roche Applied Science, Laval, Quebec, Canada): 95 °C/300 s, and 40 cycles of 95 °C/10 s, 60 °C/20 s and 70 °C/30 s. The 2^−ΔΔCT^ method was used to calculate the mRNA expression of ATG5 and ATG16L1.

### Western blotting

The treated cells were harvested and the RIPA lysis buffer (Beyotime, Shanghai, China) was used to extract the total proteins. A BCA Protein Assay Kit (Thermo) was used to detect the concentrations of the proteins according to the manufacturer’s instructions. The proteins (30 μg per lane) were separated by electrophoresis on a SDS-polyacrylamide gel and transferred to PVDF membrane (Millipore, Bedford, MA, USA). The membranes were incubated at 4 °C overnight with anti-ATG5 (rabbit polyclonal antibody, 1:200 diluted, BA3525-2, Boster Biological Technology, China) and anti-β-actin antibody (1:2000 diluted, Santa, CA, USA), followed by HRP-linked secondary antibodies, and visualized with an enhanced chemiluminescence (ECL) detection kit (Millipore, Billerica, MA, USA).

### Annexin V apoptosis assay

The ANXA5/AnnexinV-FITC Apoptosis Detection Kit (Beyotime institute of biotechnology, Shanghai, China) was used to detect the apoptosis as per the protocol of the manufacturer. Briefly, cells were collected and washed with PBS twice and resuspended in 195 μL of binding buffer. 5 μL of ANXA5-FITC stock solution and 10 μL of propidium iodide (PI) were added to the cells and incubated for 15 minutes at room temperature, protected from light. Then the cells were immediately analyzed by FACS.

### Cytokine measurements

Concentrations of human TNF-α, IL-6 and IL-1β were measured by using each specific enzyme linked immunosorbent assay (ELISA) kits (TianGen Biotech, Beijing, China), following the protocol of the manufacturer. The absorbance of samples and standards was detected at 450 nm by using a microplate reader. The minimum detectable concentrations of IL-6, IL-1β and TNF-α were 1 ng/mL, 1 pg/mL and 1 pg/mL, respectively.

### Statistical analyses

The measurement data was shown as the mean ± standard error of the mean (SEM) and compared using Student’s t-test. Genotype/allele distribution of each polymorphism was analyzed using Chi-squared test or Fisher’s exact test, and the false discovery rate-adjusted P-value was calculated by using the Bonferroni correction in multiple-time statistics. In addition, the power analyses were performed by Quanto 1.2 (University of Southern California, LA, USA), and a linkage disequilibrium (LD) map was construct to determine the extent of linkage disequilibrium between genetic variations using the Haploview (version 4.2) software (Jeffrey C Barrettand Mark J Daly, Cambridge, MA, USA). Kaplan-Meier survival analysis was performed to evaluate the association between ATG genotypes and 28-day mortality of sepsis. Statistical analysis was performed using the GraphPad Prism 4.0 (GraphPad Software Inc., San Diego, CA, USA) and SPSS version 19.0 (IBM, NY, USA). Statistical significance was defined as a P value < 0.05.

## Electronic supplementary material

Supplementary Figure S1
