# Supplementary material for: Association between genetic polymorphisms in the autophagy-related 5 gene promoter and the risk of sepsis
Source: Sci Rep. 2017 Aug 24;7:9399. doi: 10.1038/s41598-017-09978-5 (PMC5570943; doi:10.1038/s41598-017-09978-5)
Supplement: Supplementary file 1 — Supplementary Figure S1 [file 41598_2017_9978_MOESM1_ESM.pdf]

Association between genetic polymorphisms in the autophagy-related 5 gene promoter and the risk of sepsis

Author:

Yiming Shao[1#], Feng Chen[1#], Yuhua Chen[3#], Wenying Zhang[1], Yao Lin[4], Yujie Cai[2], Zihan Yin[1], Shoubao Tao[1], Qinghui Liao[3], Jianghao Zhao[2], Hui Mai[2], Yanfang He[2], Junbing He[1]*, Lili Cui[2]*

Author details:

[1]The Intensive Care Unit, Guangdong Key Laboratory of Age-Related Cardiac and Cerebral Diseases, Affiliated Hospital of Guangdong Medical University, Zhanjiang, Guangdong, PR China

[2]Institute of Neurology, Guangdong Key Laboratory of Age-Related Cardiac and Cerebral Diseases, Affiliated Hospital of Guangdong Medical University, Zhanjiang, Guangdong, PR China

[3]The Department of Endocrinology and Metabolism, Longgang District People's Hospital of Shenzhen, Shenzhen, Guangdong, PR China

[4]The Department of Stomatology, Guangdong Key Laboratory of Age-Related Cardiac and Cerebral Diseases, Affiliated Hospital of Guangdong Medical University, Zhanjiang, Guangdong, PR China

[#]These authors contributed equally to this work.

*Correspondence: junbinghe_gmc@163.com; cuilili@gdmu.edu.cn

[1]The Intensive Care Unit, Guangdong Key Laboratory of Age-Related Cardiac and Cerebral Diseases, Affiliated Hospital of Guangdong Medical University, Zhanjiang, Guangdong, PR China

[2]Institute of Neurology, Guangdong Key Laboratory of Age-Related Cardiac and Cerebral Diseases, Affiliated Hospital of Guangdong Medical University, Renmin street south 57, Xiashan district, Zhanjiang City 524001, Guangdong Province, PR China

Supplementary Figure S1

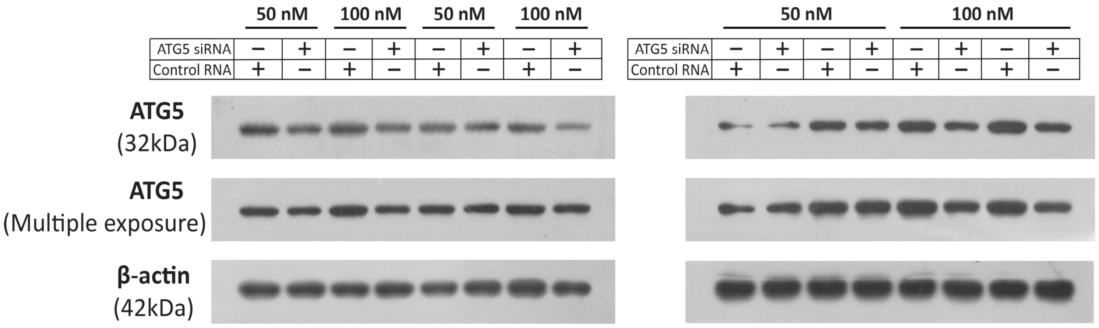

Supplementary Figure S1. The full-length and multiple exposed blots/gels. Western blotting of ATG5 in THP-1 cells transfected with ATG5 siRNA or control siRNA for 72 h.
